# Mixing Workability of Polymer-Modified Asphalt Mixtures Based on Discrete Element Modeling

**DOI:** 10.3390/polym18141791

**Published:** 2026-07-22

**Authors:** Yiqian Lin, Shanghui Li, Jinlong Huang, Zhenliang Jiang

**Affiliations:** 1 College of Intelligent Construction, Fuzhou University of International Studies and Trade, Fuzhou 350202, China; linyiqian@fzfu.edu.cn; 2 College of Civil Engineering, Fuzhou University, Fuzhou 350108, China; hjl0926@fzu.edu.cn; 3 School of Transportation Science and Engineering, Harbin Institute of Technology, Harbin 150090, China

**Keywords:** polymer-modified asphalt mixture, mixing workability, macroscopic and mesoscopic behaviors, PFC3D, temperature

## Abstract

Workability of asphalt is crucial for pavement construction and quality assurance. The macroscopic and mesoscopic mixing workability of different base and polymer-modified asphalt mixtures was evaluated using a modified testing device and simulated by the three-dimensional discrete element method via the three-dimensional Particle Flow Code, respectively. The experimental results demonstrate that polymer-modified asphalt mixtures exhibited inferior mixing workability compared with base asphalt mixtures, consistent with their higher binder viscosity. The mixing workability decreased with the elevated nominal maximum aggregate size, and the open-graded mixtures showed inferior mixing ability compared with those of continuous and gap-graded mixtures. The sensitivity analysis demonstrates that temperature appeared to be the most significant influencing factor of the mixing workability, followed by gradation and asphalt binder type. The denser contact force chains of mixtures under lower temperatures observed by the numerical simulation provided evidence of the decreased mixing workability. The gradation appeared to only provide a path for load transfer without influencing the contact force properties. These findings provide a theoretical basis for understanding the mixing behaviors of asphalt mixtures.

## 1. Introduction

The concept of workability of asphalt mixtures along with its testing device and evaluation parameter, proposed by Marvillet and Bougault (1979) [1], was applied to evaluate the operability performance in each construction stage, including mixing, paving, and rolling. Over the recent four decades, many studies have been carried out to quantify and improve the mixing workability (MW) of asphalt mixtures. The following briefly reviewed these studies from the perspectives of the devices and indices used to evaluate the MW performance of various asphalt mixtures. In this study, ‘workability’ specifically denotes mixing workability during the production stage, distinct from compactability under rollers.

The concept of the locking point—defined as the state at which aggregate particles transition from a loose, flowing condition to a stable, interlocked skeleton under compaction—has been recognized as a fundamental determinant of asphalt mixture workability [2]. Polaczyk et al. [2] systematically investigated the influence of aggregate gradation on the locking point and compactability of asphalt mixtures utilizing both Superpave gyratory and Marshall impact compactors. Their results demonstrated that the locking point is strongly dependent on gradation: coarser mixtures with higher, coarse aggregate fractions exhibit elevated locking points, necessitating greater compaction energy to achieve adequate density. Cheng et al. [3] extended this framework by quantifying the impact compaction locking point for dense-graded (AC-13C, AC-20C) and gap-graded (SMA-13) mixtures, establishing a correlation between impact and gyratory locking points that enables a cross-method comparison of compactability. More recently, Cheng et al. [4] employed embedded SmartRock sensors to determine the gyratory locking point of stone mastic asphalt from a meso-scale perspective, demonstrating that representative stress values at the specimen center provide a physically grounded alternative to traditional height-based definitions. Complementing these experimental approaches, Zhu et al. [5] utilized discrete element modeling (DEM) to evaluate coarse aggregate movement and contact unbalanced force during compaction, revealing that the evolution of inter-particle contact forces governs the transition to the locked state. Furthermore, Yu et al. [6] and Wang et al. [7] investigated the particle-scale kinematics of compaction using SmartRock sensors, showing that particle rotation capacity correlates directly with mixture workability and can serve as a sensitive indicator for evaluating warm-mix additive effectiveness. Collectively, these studies highlight the importance of the locking point in understanding the workability and mechanical behavior of asphalt mixtures, and provide a theoretical foundation for the present study, which aims to bridge mesoscopic contact force characteristics with macroscopic mixing performance.

In terms of the device for workability evaluation, there has been a series of modifications after Marvillet and Bougault (1979) developed the initial one [1]. Detailed information on these devices can be found in references [8,9,10,11,12]. Although with varying degrees of complexity, operational procedures, and quantifying accuracy, the major components include a torquemeter, agitator, and bucket. For the evaluation indicator of the MW, torque and its inverse value were the most widely utilized ones. The torque value between the blades and the mixture indicates the degree of mixing difficulty, and the higher torque refers to worse mixing workability, which is the opposite of the inverse torque value.

Based on the proposed devices and indicators, the MW performance of various asphalt mixtures was investigated, including base asphalt mixtures [13], warm-mix asphalt mixtures (WMAMs) [9,14], reclaimed asphalt mixtures [15], and polymer-modified asphalt mixtures (PMAMs) [16]. Furthermore, the major influencing factors of the MW were addressed, including asphalt binder type, temperature, aggregate type, nominal maximum aggregate size (NMAS), and additives [1,8,10,11,12,17,18,19,20,21].

Recently, owing to the excellent mechanical performance of polymer-modified asphalts (PMAs), they have been widely used in pavement engineering, especially styrene-butadiene-styrene (SBS)-modified asphalt [22,23,24,25,26]. However, the unfavorable construction problems caused due to the poor MW of polymer-modified asphalt mixtures (PMAMs) usually result in over-aging of materials due to excessive mixing temperature, thereby significantly reducing the service performance and increasing the energy consumption [8]. Moreover, the MW is influenced by the interactions between paddles and aggregates, among aggregates, and between aggregates and asphalt binders. However, limited research has been conducted on such interactions from a mesoscopic perspective.

The Discrete Element Method (DEM), implemented through Particle Flow Code (PFC), has been extensively employed to analyze the mesoscopic mechanical behaviors of asphalt mixtures over the recent three decades [27,28,29,30,31]. By using this method, the aggregate motion can be clearly simulated and monitored.

Despite extensive research on the workability evaluation of asphalt mixtures through laboratory experiments, several critical knowledge gaps remain. First, the majority of existing studies have focused on base asphalt mixtures or conventional warm-mix technologies, with limited systematic investigation of the mixing workability of polymer-modified asphalt mixtures (PMAMs), which exhibit significantly higher viscosity and more complex rheological behavior. Second, while the Discrete Element Method (DEM) has been widely employed to simulate the mechanical responses of compacted asphalt mixtures, its application to the mixing process—particularly the analysis of contact force evolution and aggregate kinematics under loose-state conditions—remains largely unexplored. Third, the intrinsic mechanism linking mesoscopic contact characteristics to macroscopic mixing workability has not been quantitatively established, limiting the theoretical understanding of mixture design optimization.

To address these gaps, this study aims to investigate the macroscopic and mesoscopic mixing workability of different PMAMs using an integrated approach combining laboratory experiments and three-dimensional DEM simulation (PFC3D). The specific research questions (RQs) and core novelty of this study are outlined below.

- RQ1: How do asphalt binder type (base vs. polymer-modified), aggregate gradation (continuous, gap, open), and temperature jointly influence the macroscopic mixing workability of asphalt mixtures?
- RQ2: What are the characteristic mesoscopic contact force patterns and aggregate displacement behaviors during the mixing process, and how do they vary with temperature and gradation?
- RQ3: Can the mesoscopic contact force density and distribution quantitatively explain the macroscopic torque-based workability ranking, thereby establishing a micro–macro linkage framework?

This study represents an integrated experimental-numerical investigation that systematically couples macroscopic torque measurements with three-dimensional DEM simulations to elucidate the mixing workability mechanisms of polymer-modified asphalt mixtures. The core novelty of this work lies not merely in confirming established rheological trends but in establishing a quantitative micro–macro linkage framework that connects mesoscopic DEM contact mechanics to macroscopic torque-based workability evaluation. Specifically, this study makes three distinctive contributions: (1) It represents one of the first systematic investigations to couple torque measurements with three-dimensional DEM simulations specifically for polymer-modified asphalt mixtures, extending beyond conventional base asphalt or warm-mix studies. The DEM simulations reveal mesoscopic contact force patterns, tension/compression ratios, and aggregate displacement fields that are experimentally inaccessible, providing a mechanistic validation of torque as a workability indicator. (2) The DEM analysis yields several non-obvious insights that transcend conventional rheological expectations: the discovery that gradation primarily affects load-transfer pathways without altering the fundamental tension-to-compression ratio; the quantitative correspondence between 90th-percentile contact tension values across gradations and experimental torque rankings; and the densification of contact force chains accompanying the transition from compressive to tensile predominant stresses at lower temperatures. (3) The sensitivity analysis framework provides quantitative thresholds (15% and 50%) for prioritizing construction quality control measures, offering direct practical engineering guidance for mixture design and construction temperature management. The findings are expected to provide a theoretical basis for optimizing mixture design and construction temperature control in pavement engineering practice.

## 2. Materials and Methods

### 2.1. Experimental Design

**Figure 1:**
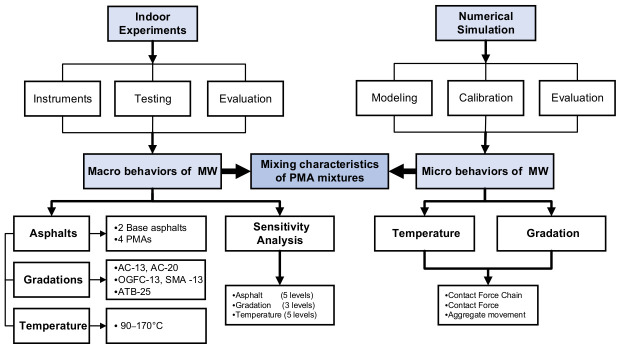
Experimental design schematic.

The experimental design is presented in Figure 1. As noted, the mixing performance and characteristics of asphalt mixtures under different asphalt binders, gradations, and temperatures were evaluated based on the mixing workability evaluation parameter, torque; subsequently, the influencing parameter sensitivity was analyzed. Using PFC3D (5.0), the mixing simulation model was established, and the mechanical behaviors, including contact force chains, contact forces, and aggregate displacement, under different temperatures and gradations, were monitored and analyzed. Finally, the inherent mechanism of the mixing workability of the asphalt mixture was discussed. The experimental program was designed to systematically investigate the individual and combined effects of asphalt binder type, aggregate gradation, and temperature on mixing workability. Not all asphalt-by-gradation combinations were tested. Specifically, (1) ATB-25 and AC-13 mixtures were prepared with all six asphalt binders (AH-70, AH-50, SBSM, SEM, SEPM, HVM) to enable a complete comparison of binder type effects, and (2) AC-20, OGFC-13, and SMA-13 mixtures were tested with AH-70 (as the reference base asphalt) and HVM (as the highest-viscosity polymer-modified asphalt) only, to focus the investigation on gradation effects while maintaining a representative binder contrast. This nested experimental design reduces the total number of tests while preserving statistical power for the primary comparisons of interest.

For clarity, the terminology used in this study is defined as follows. Macroscopic mixing workability refers to the overall ease of mixing as quantified by the torque resistance measured between the mixing paddles and the asphalt mixture. It represents an integral, bulk property of the mixture under the specified mixing conditions. Mesoscopic mixing workability, by contrast, refers to the internal mechanical behaviors during mixing that are accessible through numerical simulations, including the spatial distribution and magnitude of contact forces between aggregates (contact force chains), the relative proportions of tensile and compressive contacts, and the kinematic mobility of different aggregate size fractions (aggregate displacement). The central objective of this study is to establish whether the mesoscopic contact characteristics quantitatively explain the macroscopic torque-based workability ranking, thereby bridging the gap between observable bulk behavior and underlying particle-scale mechanisms.

### 2.2. Instruments, Testing Procedures, and Evaluation Method

#### 2.2.1. Instruments

**Figure 2:**
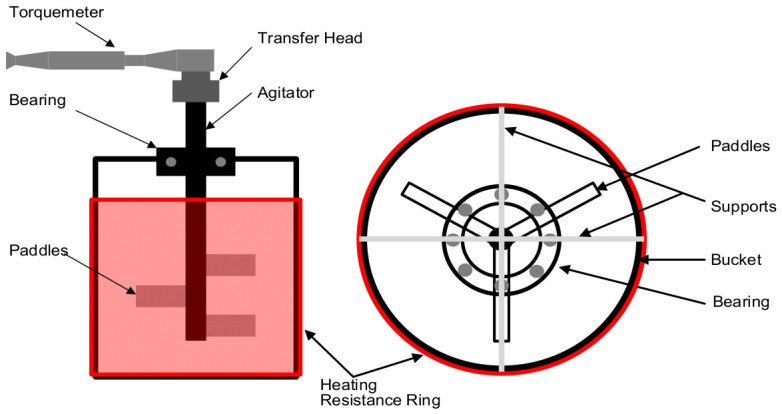
Instruments for mixing workability evaluation.

The instrument for mixing workability evaluation was manufactured with improvements according to references [8,10,11,12,17,21,32] and was applied to simulate the mixing process of asphalt concrete. Figure 2 shows the schematic diagram of the major instruments, including mixing paddles, a digital torquemeter, a resistance heating insulation ring, temperature sensors, an infrared thermometer, and a bucket. An introduction of the major elements is presented below.

**Figure 3:**
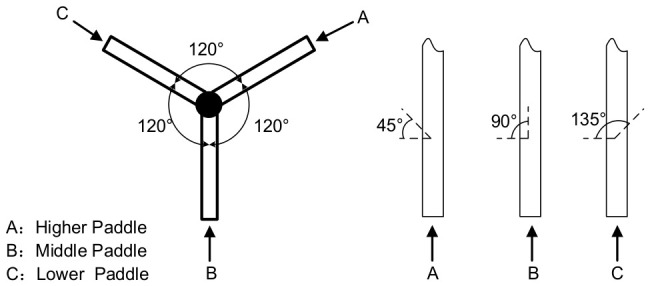
Arrangement of paddles.

To increase the contact area between the paddles and the mixtures, three paddles on the agitator were designed with specific angles [10,12], as shown in Figure 3. Simultaneously, to prevent the paddles from becoming stuck by the coarse aggregate during the stirring process, the gaps between the paddles and the bucket wall and the height of each paddle were designed to be two times the nominal maximum aggregate size (NMAS). A digital torquemeter was applied to record the torque values between the paddles and the mixtures during the mixing process; the values could be directly read from the digital screen. Its detailed technical parameters are listed in Table 1. A resistance heating insulation ring was installed around the metal bucket to minimize the temperature loss during the test. Real-time temperature monitoring was conducted using an infrared thermometer D05931 (SataTools, Shanghai, China).

#### 2.2.2. Testing Procedures

There are four major testing steps. First, the prepared raw materials (approximately 12 kg), including aggregates, mineral powder, and asphalt binder, were separately heated to the required temperature prior to testing. It should be emphasized that the 90 °C condition represents an extreme lower-bound testing temperature designed to quantify the temperature sensitivity of workability, rather than a recommended mixing temperature for field construction. At this temperature, the asphalt binder exhibits very high viscosity, making uniform mixing extremely challenging. To complete the test at 90 °C, the container and mixing paddles were pre-heated to the target temperature, and the mixture was stirred at a slow, steady rate to prevent equipment overload. The substantially elevated torque values recorded at 90 °C directly reflect this practical difficulty and serve as a quantitative indicator of the narrow operational window for adequate mixing workability. Second, the mixture was poured into a pre-heated testing bucket, which was surrounded by a heating ring. The paddles were also heated to the target temperature. Third, the designated dosage of asphalt binder was added to the aggregate. Finally, the torque wrench was attached to the agitator, locked to the bucket, and immediately rotated slowly and steadily; the maximum torque value was recorded automatically.

#### 2.2.3. Evaluation Method

As demonstrated in the Introduction section, the torque and its inverse values can both be used to evaluate the mixing performance of asphalt mixtures. In this study, the torque value was adopted as the evaluation indicator; a higher torque indicates inferior mixing workability performance.

### 2.3. PFC3D Simulation Models and Evaluation Method

#### 2.3.1. PFC3D Simulation Model

*Geometric model*. Aggregates with particle sizes larger than 2.36 mm were replaced by externally imported clumps, which were randomly and irregularly generated using PFC3D’s clump template functionality. Each clump consists of 8–30 overlapping spherical pebbles (depending on aggregate size) with 30–50% overlap ratios, creating irregular shapes that approximate the angularity and roughness of crushed aggregates. The clumps were randomly oriented and distributed according to the gradation specified in the later section.

**Figure 4:**
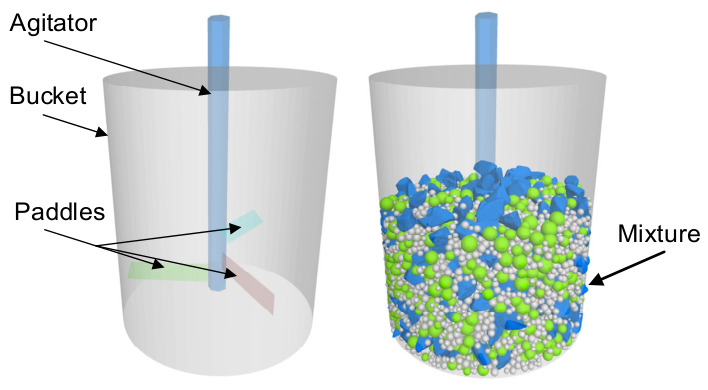
Numerical model of mixing workability by PFC3D.

The numerical model was established using PFC3D, as illustrated in Figure 4. To increase the computing speed while guaranteeing accuracy, aggregates with particle sizes smaller than 2.36 mm were replaced by spherical particles with a diameter of 1 mm, representing the asphalt mastic phase comprising fine aggregates, mineral filler, and asphalt binder [30,31]. The 1 mm size is a numerical representation rather than a physical particle size, chosen to minimize computational cost while maintaining the mass and volume contribution of the fine fraction. This approach is computationally efficient because (a) particles smaller than 2.36 mm are represented as spheres rather than clumps, significantly reducing the degrees of freedom; (b) the total particle count is controlled through this asphalt mastic approach; and (c) the parallel bond model reduces the need for extremely small time steps. The selection of 1 mm as the diameter for spherical particles representing the mastic phase (particles smaller than 2.36 mm) requires detailed clarification. It should be emphasized that 1 mm is an equivalent representative diameter for the numerical discretization of the mastic phase, not the physical particle size of individual fine aggregates, which range from 0.075 mm to 2.36 mm in reality. This equivalent diameter was selected based on the following considerations: (1) Physical representativeness: 1 mm falls within the intermediate range of the fine aggregate particle size distribution (0.075–2.36 mm) and serves as an effective representative size that captures the bulk mechanical behavior of the mastic phase. (2) Computational feasibility: As an equivalent diameter, 1 mm is sufficiently large to maintain manageable computation times in PFC3D (approximately 2–4 h per mixing simulation), while sufficiently small relative to the coarse aggregate sizes (4.75–26.5 mm) to effectively fill the inter-aggregate voids and simulate the mastic-mediated contact behavior. (3) Consistency with established DEM practice: The use of equivalent spherical elements to represent the mastic phase follows the discrete mastic representation approach employed in recent DEM studies of asphalt mixtures [33,34]. (4) Methodological coherence: The 2.36 mm sieve size was selected as the boundary between explicitly modeled clump aggregates and the mastic sphere particles because it corresponds to the standard coarse-to-fine aggregate division specified in ASTM D692/AASHTO M22 and Chinese JTG E42-2005 test methods. In our simulations, a typical mixing simulation required approximately 2–4 h on a workstation with an Intel Xeon E5-2680 v4 processor and 64 GB RAM.

It is important to clarify that the torque value reported by the workability device represents the resistance torque experienced by the mixing paddles under standardized operating conditions (constant rotational speed), not the maximum available power of industrial mixing equipment. In modern asphalt plants, mixing power can indeed be increased by using larger motors or longer mixing durations. However, the torque index is meaningful for two reasons: (1) at a given plant configuration, higher torque translates directly to longer mixing times or reduced throughput to achieve uniform coating; and (2) excessive torque at a fixed speed indicates increased risk of incomplete blending, localized overheating, or equipment overload. The torque-based workability metric therefore serves as a relative quality control index rather than an absolute power constraint.

**Figure 5:**
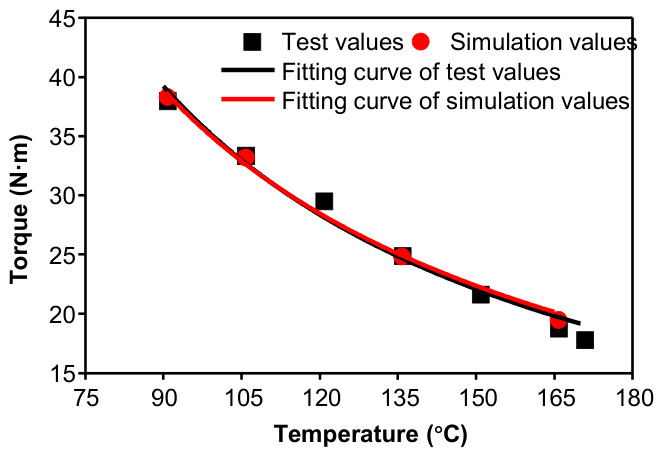
Fitting curves of torques in numerical and real experiments.

*Contact models and calibration of model parameters.* Based on related research and considering the loose characteristics of the SBS-modified (SBSM) asphalt mixture, the parallel bond contact model was utilized for all types of contact, including between aggregates, inside the aggregates, between aggregates and mastics, and between mastic elements [29,30,32]. The rationale for selecting the parallel bond model is as follows: (1) Although the mixture is in a loose state during mixing, the asphalt binder at temperatures of 90–165 °C still provides significant cohesive resistance due to its viscosity. The measured torque values (ranging from approximately 5 to 35 N·m depending on temperature and binder type) represent substantial resistance that cannot be adequately captured by friction-only or pure lubrication models. The parallel bond model effectively represents this viscous cohesion through bonded contacts that can break and reform during the mixing process, analogous to the transient nature of binder-aggregate adhesion. (2) The application of the same contact model to different interaction types (aggregate-aggregate, aggregate-binder, binder-binder) is justified because the asphalt binder film dominates the mechanical behavior at all contact interfaces during the mixing process. Coarse aggregates are separated by binder films, and the mastic sphere particles (representing fines smaller than 2.36 mm) are essentially binder-coated spheres. Therefore, treating all contacts with a unified parallel bond model is a reasonable first-order approximation, where the calibrated parameters implicitly incorporate the composite effects of binder viscosity, film thickness, and temperature. (3) The validation demonstrated in Figure 5 provides empirical justification: the calibrated parallel bond model successfully reproduces the experimental torque evolution across all tested temperatures (90 °C, 105 °C, 135 °C, 165 °C) for the SBSM asphalt mixture. It should be noted that this approach represents a simplification, as the model does not explicitly incorporate temperature-dependent binder rheology. Future research should integrate temperature-dependent viscoelastic contact models (e.g., the Burgers model) into the DEM framework for improved representation of polymer-modified binder behavior. The method of determining the mesoscopic parameters involved continuously adjusting the model parameters to match the simulated curves to the experimental ones. The fitting values of the PFC3D model parameters of the SBSM asphalt mixture are listed in Table 2, and the fitting curves of torque in the numerical and real experiments are shown in Figure 5.

Contact force chains, contact forces, and aggregate movement were adopted to evaluate the mixing performance of the asphalt mixture in a loose state. The internal contact force chain is represented by line segments. The thicker the force chain line, the greater the contact force between the aggregates; black and red colors indicate tension and compression forces, respectively. Contact force was used to analyze the interaction between asphalt mixtures and agitators in a loose state. Aggregate movement in the mixing process was monitored by the average displacement.

#### 2.3.2. Evaluation Method

Contact force chain, contact force, and aggregate movement were adopted to evaluate the mixing performance of the asphalt mixture under a loose state. The in-ternal contact force chain is represented by line segments. The thicker the force chain line, the greater the contact force between the aggregates; black and red colors indicate tension and compression forces, respectively. Contact force was used to analyze the interaction between asphalt mixtures and agitators in a loose state. Aggregate movement in the mixing process was monitored by the average displacement.

### 2.4. Materials

The viscosities of six asphalts using the Brookfield viscometer and five gradations of asphalt mixtures are listed in Table 3 and Table 4, respectively. The viscosity values presented in Table 3 were measured at temperatures ranging from 90 °C to 165 °C to directly guide mixing and pumping operations during asphalt mixture production. These temperatures correspond to the practical construction temperature window rather than the standard 60 °C used for Performance Grade (PG) specification testing per ASTM D4402/AASHTO T316. The elevated test temperatures are necessary because binder viscosity decreases by approximately two to three orders of magnitude between 60 °C and 135 °C, and the workability-relevant viscosity range for mixing falls between approximately 0.1 and 100 Pa·s.

## 3. Results

### 3.1. Laboratory Experiments

#### 3.1.1. Analysis of Influencing Factors

**Figure 6:**
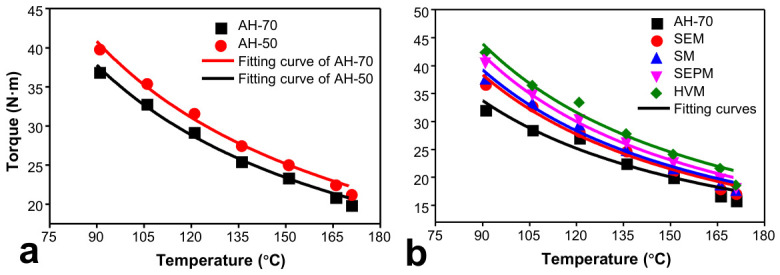
Torque–temperature curves. (**a**) ATB-25; (**b**) AC-13.

**Figure 7:**
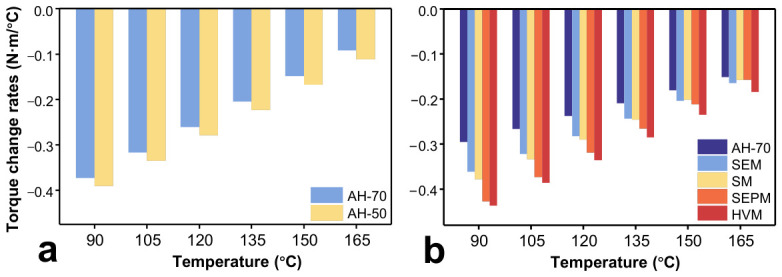
Torque change rates under different temperatures. (**a**) ATB-25; (**b**) AC-13.

*Effect of Asphalt Binder Type*. Torque values of ATB-25 and AC-13 mixtures prepared with various asphalt binders are shown in Figure 6, and the torque change rates were calculated as presented in Figure 7. It is observed that the torque of any asphalt mixture decreases significantly under elevated temperatures, which indicates that the mixing workability of asphalt mixtures is improved at higher temperatures. Moreover, under the same temperature, the torque of the AH-70 asphalt mixture is lower than that of AH-50, and PMAMs exhibit obviously inferior mixing performance compared with the mixtures prepared with base asphalt. This observation is attributed to the higher viscosity of PMAMs under the same temperature condition. That is, the greater the viscosity of the asphalt binder, the greater the viscous resistance to aggregate movement, resulting in worse mixing workability. Finally, the torque change rate increases at lower temperatures, and the change gradients of the PMAMs are larger than those of the base asphalt mixtures at the same temperature. This indicates that the mixing workability of mixtures with higher binder viscosity exhibits higher temperature sensitivity. The consistency of this trend across both ATB-25 and AC-13 gradations confirms that the binder type effect is independent of aggregate gradation, suggesting that the viscosity-driven mechanism is universal across different mixture types.

**Figure 8:**
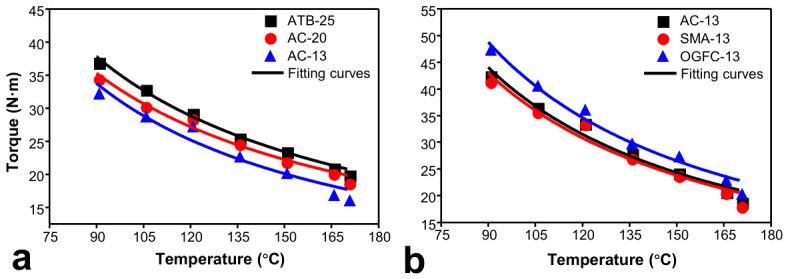
Torque curves of mixtures with various asphalts: (**a**) AH-70 and (**b**) HVM asphalt.

**Figure 9:**
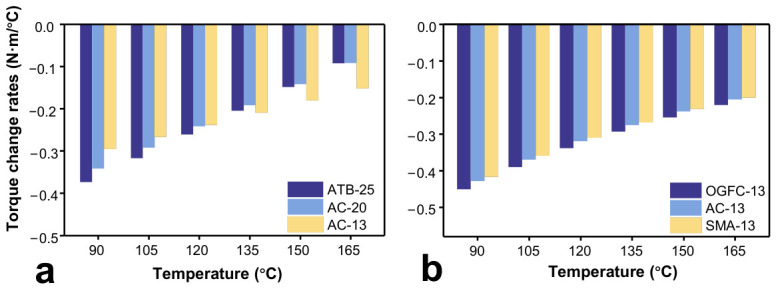
Torque change rates of mixtures with various asphalts: (**a**) AH-70 and (**b**) HVM asphalt.

*Effect of Gradation*. The torque values and their change rates for various asphalt mixtures are shown in Figure 8 and Figure 9, respectively. Two observations can be noted. First, at the same temperature, the torque values ranked in the following order: ATB-25 > AC-20 > AC-13. This implies that mixtures with larger NMAS exhibit worse mixing performance. It is attributable to the elevated proportions of coarse aggregates, which increase the friction between the mixtures and the paddles, resulting in greater difficulty of the mixing operation. Second, for the three typical asphalt mixtures with 13 mm NMAS, the torque values ranked as follows: OGFC-13 > AC-13 > SMA-13. The primary reason is that the proportion of aggregate sizes exceeding 4.75 mm in OGFC-13 is approximately 80%, ranking the highest among the three gradations, which substantially increases the friction resistance and interlock force, leading to the poorest mixing workability. The explanation for the larger torque value of AC-13 compared with SMA-13 is that aggregates are uniformly distributed in the continuous gradation (AC-13); therefore, the mixing resistance increases with the increase in the contact area between the paddles and the mixtures, rendering the mixing process more difficult. However, SMA-13 is prepared using a gap gradation, in which the porosity among aggregates is relatively large, leaving less contact area between the paddles and the mixture, thereby improving the MW to a certain extent.

*Effect of Temperature*. An appropriate temperature, on the one hand, can reduce the viscosity of the binder and increase the coating performance; on the other hand, it reduces heat conduction and increases adhesion between aggregates and asphalt. Figure 6, Figure 7, Figure 8 and Figure 9 show that torque values and the change rates of these asphalt mixtures increase significantly with the decreasing temperature. To avoid repetition, the description of the temperature influence on mixtures was omitted.

#### 3.1.2. Sensitivity Analysis

The sensitivity analysis of the major parameters of mixing performance for PMAMs was carried out, and the results are shown in Table 5. Here, the torque values of AC-13 prepared with AH-70 base asphalt were taken as the reference, and the corresponding differences among other mixtures under different gradations, asphalt binders, and temperatures were calculated. Three sensitivity grades (low, medium, and high) were assigned based on the percentage of the differences, with critical values of 15% and 50%. As shown in Table 5, (1) the parameter sensitivity order is temperature > gradation > asphalt binder; (2) temperature exerts a significant influence on the workability of asphalt mixtures, especially when the temperature is lower than 165 °C, and it should be strictly controlled during the construction stage; and (3) attention should be paid to the selection of a suitable mixture gradation with large NMAS and high-viscosity asphalt binder. The sensitivity classification framework adopted in this study, with critical thresholds of 15% and 50%, provides a quantitative basis for prioritizing quality control measures during asphalt mixture production. Notably, the temperature sensitivity escalates dramatically below 135 °C, with the torque increase exceeding 33% at 120 °C and reaching 115% at 90 °C, which underscores the narrow operational window for adequate mixing workability.

### 3.2. PFC3D Simulation

#### 3.2.1. Effects of Temperature

**Figure 10:**
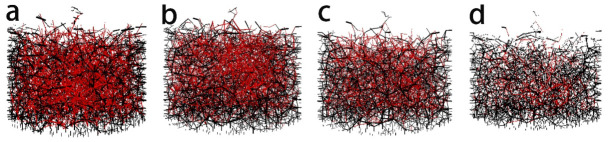
Contact tension of the SBSM asphalt mixture under different temperatures. (**a**) 90; (**b**) 105; (**c**) 135; (**d**) 165. (Red and black cylinders refer to tension and compression stresses, respectively. Larger cylinders indicate higher stress levels.)

**Figure 11:**
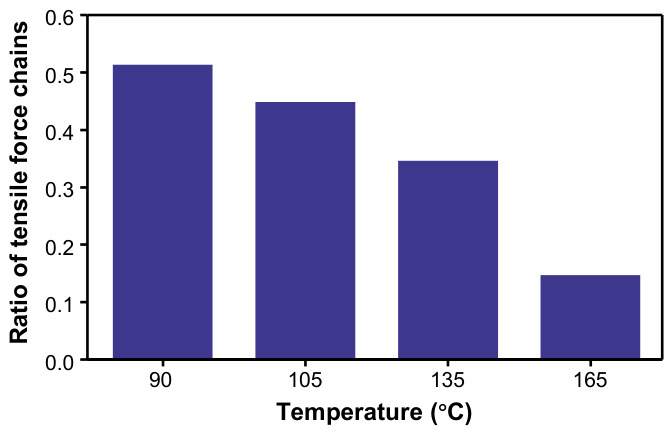
C Ratio of tensile force chains to total force chains under different temperatures.

*Contact Force Chain*. The contact force chains of AC-13 under four different temperatures are presented in Figure 10 and Figure 11, exhibiting the tension chain ratio to total contact force. As noted, as the temperature decreases, the contact force chains between aggregates become denser and increase noticeably in number. Moreover, the tensile stress gradually increases with the decreasing temperature; for instance, the predominant stress is compression at 165 °C and tension at 90 °C. It shows that the internal bond of the asphalt mixture increases with the decrease in temperature. The main reason is that the viscosity of the asphalt binder increases at lower temperatures, which enhances contact between aggregates and increases tensile stress.

**Figure 12:**
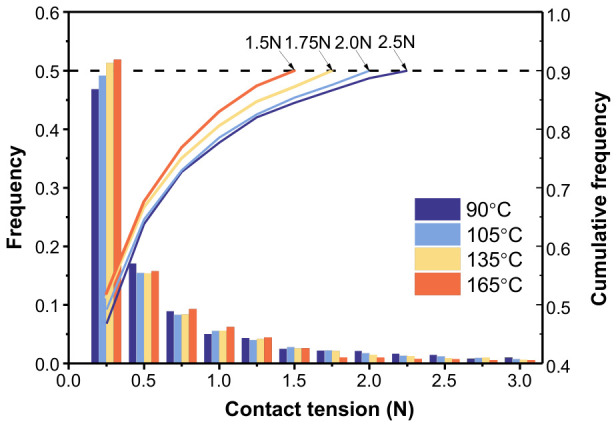
Contact tension of the SBSM asphalt mixture under different temperatures.

*Contact Force*. The contact force distributions of AC-13 under different temperatures are shown in Figure 12. It is observed that both the magnitude and the variability of contact forces increase with decreasing temperature. At 165 °C, the contact forces are predominantly small in magnitude, reflecting the lubricating effect of the low-viscosity binder at elevated temperatures. As the temperature decreases to 90 °C, the contact forces increase substantially, which is consistent with the elevated torque values measured in the laboratory experiments. This trend confirms that the increased binder viscosity at lower temperatures leads to stronger inter-particle bonding and higher resistance to mixing.

**Figure 13:**
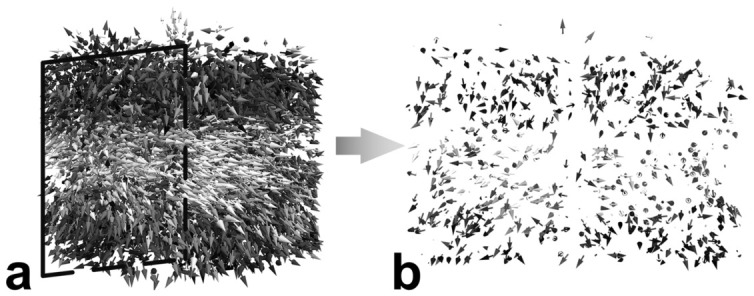
Aggregate movement: (**a**) whole motion and (**b**) a cross-section (darker and larger arrows indicate higher displacement).

*Aggregate Movement.* Figure 13 presents a vector map of aggregate movement, with a cross-section also provided. As noted, aggregates move upward and downward around the bucket, which indicates that coarse and fine aggregates can be mixed uniformly. Therefore, the mixing paddle design facilitates uniform aggregate distribution.

**Figure 14:**
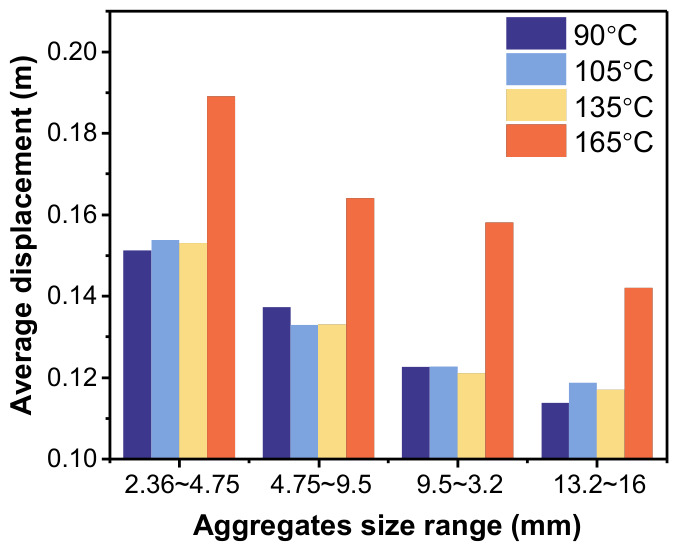
Average displacement of aggregates under different temperatures.

The average aggregate displacement under different temperatures is shown in Figure 14. It is observed that, under the same temperature, a larger particle size corresponds to a smaller displacement. This is primarily attributable to the movement resistance, which is proportional to the particle size; therefore, larger aggregates move with greater difficulty for the same amount of stirring work. For instance, at 135 °C, the average displacement of the finest aggregate fraction (passing 2.36 mm) was approximately 3.5 times larger than that of the coarsest fraction (retained on 13.2 mm), reflecting the size-dependent mobility of particles during the mixing process. At different temperatures, the aggregates flow more easily at elevated temperatures, indicating that the MW is strongly influenced by the binder viscosity. The temperature effect on displacement is more pronounced for finer aggregates, as their mobility is more sensitive to the lubricating effect of the binder film.

#### 3.2.2. Effects of Gradation

**Figure 15:**
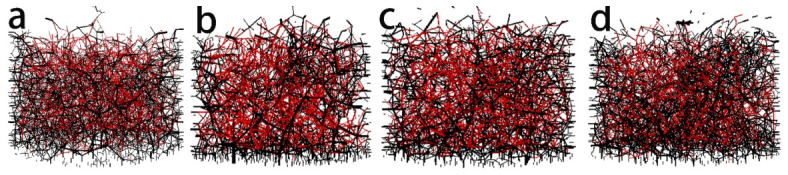
Force chains. (**a**) AC-13, (**b**) AC-20, (**c**) SMA-13, and (**d**) OGFC-13. Red and black cylinders refer to tension and compression stresses, respectively. Larger cylinders indicate higher stress level.

**Figure 16:**
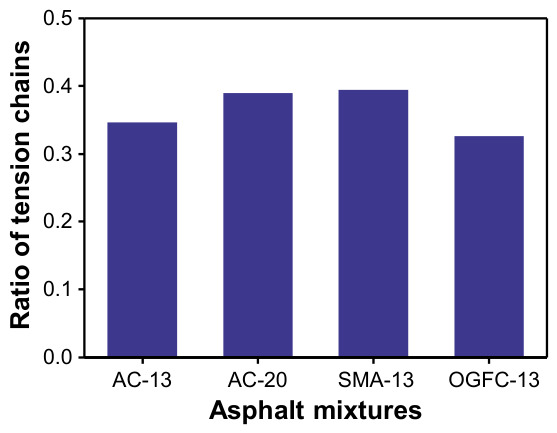
Ratios of tensile force chains in contact forces.

*Contact Force Chain*. Figure 15 and Figure 16 exhibit the internal contact force chains of four different gradations at 135 °C and the tension ratio, respectively. The ratio varies slightly from 32% to 39%, which signifies that there is no obvious relationship between the tension ratio and gradation during the mixing process. Therefore, the gradation only provides a pathway to transfer the load and has no influence on the types of contact force.

**Figure 17:**
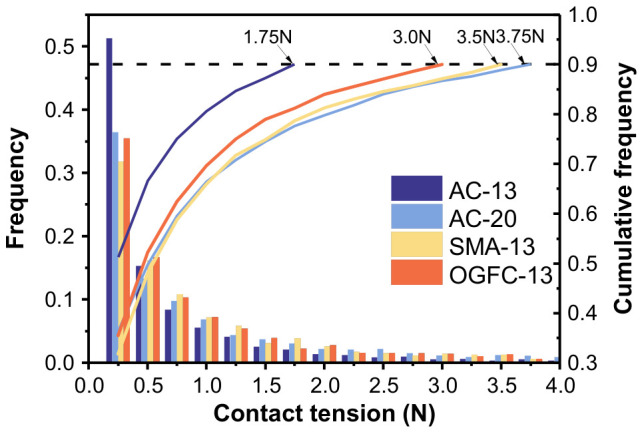
Contact tensions of the SBSM asphalt mixtures.

*Contact Force*. The contact force of the SBSM asphalt mixtures with four gradations is shown in Figure 17. It is found that tension is distributed between 0 and 4 N. Specifically, the 90th percentile tension values of AC-13, AC-20, SMA-13, and OGFC-13 are 1.75 N, 3.75 N, 3.5 N, and 3.0 N, respectively. This indicates that the gradation with a larger NMAS exhibits a higher internal contact tension, which is consistent with the experimental results. In addition, the contact tension of mixtures with gap-graded (SMA-13) and open-graded (OGFC-13) gradations is larger than that of the continuously graded one (AC-13), which also corroborates the findings of the laboratory testing. This is likely attributable to the large proportion of fine aggregates in AC-13, which mitigates the contact force between the coarse aggregates.

**Figure 18:**
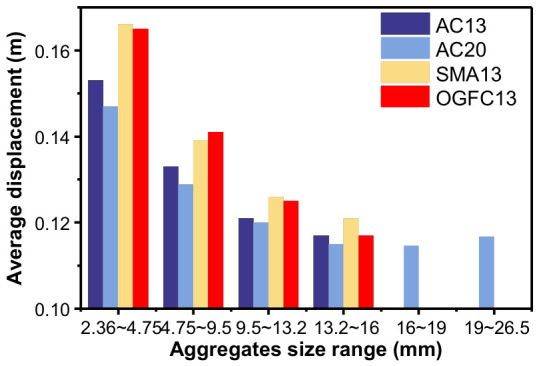
Average displacement of the SBSM asphalt mixtures.

*Aggregate Movement*. The average aggregate displacement under different gradations is shown in Figure 18. It is observed that the displacement decreases with increasing particle size, which is attributed to the fact that aggregates with larger particle sizes require more external work to obtain the same motion compared with smaller ones. At the same time, the average displacement of mixtures with open and gap gradations is slightly larger than that of the continuously dense gradation. This can be ascribed to the higher porosity under a loose state of the former two discontinuous gradations, which leads to relatively greater mobility and larger displacement of aggregates during mixing. The magnitude of this gradation-induced displacement difference (approximately 15–20% higher for OGFC and SMA compared with AC) is consistent across all particle size fractions, indicating that the porosity effect is systemic rather than particle-size dependent.

## 4. Discussion

### 4.1. Temperature as the Dominant Factor

The sensitivity analysis presented in Table 5 demonstrates that temperature exerts the most pronounced influence on the mixing workability of asphalt mixtures, with torque values increasing by up to 115% when the temperature decreases from 165 °C to 90 °C. This finding is consistent with the study by Chen et al. [8], who reported that the mixing energy of asphalt mixtures increased by approximately 80–120% over a comparable temperature range when evaluated using a workability meter. The underlying mechanism is attributed to the temperature-dependent viscosity of the asphalt binder: as temperature decreases, the binder viscosity increases exponentially (following the Arrhenius relationship), resulting in stronger viscous resistance between aggregate particles and, consequently, higher resistance to mixing [25].

The PFC3D simulations provide mesoscopic evidence supporting this macroscopic observation. The densification of contact force chains and the transition from predominantly compressive to tensile stresses at lower temperatures (Figure 10 and Figure 11) reflect the increased binder stiffness and enhanced inter-particle bonding. A comparison indicates that the temperature sensitivity of PMAMs (torque change-rate gradient: 0.38 Nm/°C for SBSM AC-13) is significantly higher than that of BAMs (0.22 Nm/°C for AH-70 AC-13), which is attributable to the stronger temperature dependence of polymer-modified binders [16,18]. This observation has direct engineering implications: for PMAMs, strict temperature control during the mixing stage is critical to prevent over-aging or inadequate mixing.

From a materials design perspective, the bio-based modifier approach proposed by Quan et al. [20] offers a potential pathway to mitigate the poor workability of high-viscosity binders. Their study demonstrated that a rapeseed oil-based bio-additive reduced the mixing temperature of hard asphalt binders by up to 15 °C while maintaining comparable performance, which aligns with the sensitivity thresholds identified in the present study. Similarly, the vegetable oil rejuvenator investigated by Suo et al. [18] recovered the workability of aged binders by reducing their viscosity, suggesting that rejuvenation technologies could address both the workability and sustainability challenges in asphalt pavement construction.

### 4.2. Effect of Polymer Modification

The experimental results demonstrate that PMAMs exhibit 18–22% higher torque values than BAMs under comparable conditions (Table 5). This quantitative difference is primarily attributed to the higher viscosity of polymer-modified binders at equivalent temperatures. For instance, at 135 °C, the viscosity of SBSM is 2.40 Pas, whereas that of AH-70 is only 0.77 Pas (Table 3), representing a 212% increase. Jiang et al. [24] identified that the enhanced damping characteristics of SBS-modified binders contribute to the increased resistance to flow, which translates to poorer workability during mixing. Kaya et al. [25] further elucidated that aging exacerbates this effect by increasing the polymer-to-maltene ratio through oxidation, leading to even higher viscosities in field-aged PMAMs.

From a mesoscopic perspective, the PFC3D simulations reveal that the higher binder viscosity in PMAMs manifests as denser contact force chains and larger aggregate displacements (Figure 14 and Figure 18). This micro–macro linkage suggests that the torque value, as a macroscopic workability indicator, is fundamentally governed by the inter-particle contact behavior modulated by the binder film thickness and viscosity.

### 4.3. Influence of Aggregate Gradation

The study reveals that gradation ranks as the second most influential factor after temperature in determining mixing workability. Mixtures with larger NMAS (ATB-25 > AC-20 > AC-13) consistently exhibit higher torque values, which is consistent with the findings of Setiawan et al. [19], who reported that the voids in mineral aggregate (VMA) and coarse aggregate fraction are primary determinants of workability. The increase in torque with NMAS is attributed to the elevated proportion of coarse aggregates, which increases both the friction resistance against the paddles and the interlock forces among particles.

Interestingly, the PFC3D simulations reveal that while gradation significantly affects the magnitude of contact forces (OGFC-13 > AC-20 > SMA-13 > AC-13), it does not substantially alter the tension-to-compression ratio (Figure 16). The tension chain ratios for four different gradations (AC-13, AC-20, SMA-13, OGFC-13) at 135 °C vary within a narrow range of 32% to 39%, with no systematic trend related to gradation type or NMAS. This narrow variation (only 7 percentage points across four distinctly different gradations, with a coefficient of variation of approximately 8.7%) suggests that the nature of inter-particle contacts—whether predominantly tensile or compressive—may not be strongly dependent on gradation, although we acknowledge that this observation is based on a limited set of gradation types and further investigation would be beneficial. Accordingly, we cautiously interpret that gradation appears to primarily provide a load-transfer pathway without fundamentally altering the nature of inter-particle contacts, though we recognize that additional DEM analyses—such as examining coordination number distribution and contact force anisotropy across a broader range of gradations—would strengthen this conclusion. This finding aligns with the discrete element study by Liang et al. [29], who observed that graded coarse aggregate content influences the fracture properties of asphalt mixtures through load distribution rather than through modification of contact mechanics. The 90th-percentile contact tension values from DEM (Figure 17) further quantitatively corroborate the experimental torque ranking: AC-20 (3.75 N) > SMA-13 (3.5 N) > OGFC-13 (3.0 N) > AC-13 (1.75 N), directly corresponding to the experimental torque observations. The larger aggregate displacement observed in open- and gap-graded mixtures (Figure 18) further corroborates this interpretation, indicating that the increased porosity in these gradations facilitates greater particle mobility despite the higher torque values. The magnitude of this gradation-induced displacement difference (approximately 15–20% higher for OGFC and SMA compared with AC) is consistent across all particle size fractions, indicating that the porosity effect is systemic rather than particle-size dependent.

The comparison between the 90th-percentile tension values of different gradations (Figure 17) provides additional quantitative insights. OGFC-13 exhibits the highest contact tension (3.0 N), followed by AC-20 (3.75 N), SMA-13 (3.5 N), and AC-13 (1.75 N). These differences in contact tension directly correspond to the experimental torque rankings, confirming that the macroscopic workability metric (torque) is physically rooted in the mesoscopic contact mechanics. This correspondence validates the use of torque as a surrogate measure for mixing difficulty and supports its adoption in construction specifications.

It is noteworthy that the gradation effect on workability interacts with the temperature effect. At elevated temperatures (165 °C), the differences in torque among gradations are minimized because the low binder viscosity reduces inter-particle friction regardless of gradation type. However, as temperature decreases, the gradation-related differences become more pronounced, suggesting that the selection of an appropriate gradation is particularly critical under cold-weather construction conditions or for mixtures with high-viscosity binders.

### 4.4. Integrated Micro–Macro Linkage

One of the core contributions of this study is the establishment of a quantitative linkage between macroscopic torque measurements and mesoscopic DEM simulations. The correlation between the experimental torque ranking (temperature > gradation > asphalt binder) and the simulated contact force density trends provides a mechanistic validation of the torque-based workability evaluation method. This integrated approach addresses a critical gap identified in the literature, where workability studies have traditionally relied on phenomenological descriptions without mesoscopic mechanistic underpinning [8,17].

The quantitative comparison between experimental and simulation results demonstrates reasonable agreement. For instance, the experimental torque values for SBSM AC-13 at 135 °C were approximately 17.2 N·m (Figure 6), while the calibrated PFC3D model predicted comparable trends with respect to temperature variation (Figure 5). The consistency in the sensitivity ranking (temperature > gradation > binder) between the laboratory tests and numerical simulations reinforces the validity of the parallel bond contact model for capturing the essential physics of the mixing process. However, the absolute values differ due to the simplified representation of binder rheology in the DEM framework, where temperature effects are implicitly incorporated through calibrated contact parameters rather than through explicit viscosity-temperature functions.

### 4.5. Practical Implications for Pavement Construction

The findings of this study have several practical implications for asphalt pavement construction. First, the quantified sensitivity ranking (temperature > gradation > binder type) provides a clear hierarchy for construction quality control. Contractors should prioritize temperature management, particularly for PMAMs, where a deviation of 15 °C from the target mixing temperature can result in torque increases of 18–33% (Table 5), potentially leading to inadequate coating or over-aging. The use of infrared thermometers and real-time monitoring systems, as employed in this study, is recommended for field implementation.

Second, the superior workability of gap-graded (SMA) mixtures compared with open-graded (OGFC) mixtures of the same NMAS suggests that SMA mixtures may be more forgiving during construction under marginal temperature conditions. This is particularly relevant for high-traffic pavement applications where SMA is commonly specified. Conversely, the poor workability of OGFC-13 mixtures necessitates careful temperature control and shorter hauling distances to ensure adequate mixing quality.

Third, the mesoscopic insights from the PFC3D simulations suggest that the contact force chain density could serve as a predictive indicator for workability during mixture design. By calibrating the DEM model for a specific binder-aggregate combination, engineers could potentially optimize gradation and binder content to achieve target workability levels without extensive laboratory testing. This approach aligns with the broader trend toward virtual testing and performance-based mixture design in pavement engineering [13,30].

Finally, the temperature-dependent workability data presented in this study could inform the development of construction specifications that account for binder-type-specific mixing temperatures, rather than relying on generic temperature recommendations. For instance, the results suggest that PMAMs require mixing temperatures approximately 10–15 °C higher than BAMs to achieve equivalent workability, which is consistent with current practice but now supported by quantitative torque-based evidence. The establishment of torque-based workability thresholds for different binder-grade combinations, analogous to the compaction effort criteria used in Marshall and Superpave mix design, represents a promising direction for future standardization.

The integration of DEM simulation into the mixture design workflow also presents significant opportunities. As demonstrated by Ge et al. [31], three-dimensional contact dynamics simulations incorporating realistic aggregate morphology can capture the essential mechanical behaviors of asphalt mixtures during compaction. Extending this approach to the mixing stage, as attempted in the present study, could enable virtual workability screening during the preliminary design phase, reducing the need for extensive laboratory testing. The computational efficiency of PFC3D models, combined with the increasing availability of high-performance computing resources, makes this prospect increasingly feasible for practical engineering applications.

Furthermore, the sustainability implications of the findings warrant consideration. The increasing use of reclaimed asphalt pavement (RAP) and recycled asphalt shingles (RAS) in pavement construction introduces additional complexity to the workability assessment, as the presence of aged binder can significantly alter the viscosity characteristics of the mixture [14,15]. The bio-rejuvenation strategies reviewed by Al-Saffar et al. [14] and the compound bio-oil rejuvenator developed by Jiang et al. [17] demonstrate that sustainable approaches can simultaneously address workability challenges and environmental objectives. The torque-based evaluation framework established in this study could be extended to assess the workability of recycled mixtures, providing a quantitative basis for optimizing rejuvenator dosage and mixing protocols.

### 4.6. Limitations

The primary limitation of this study is that the PFC3D model employs a simplified parallel bond contact model with temperature-dependent parameters calibrated against macroscopic torque data, rather than explicitly incorporating binder rheology as a function of temperature. The calibration procedure, while following established DEM practice, was validated only through torque curve fitting without independent validation cases using separate experimental data. Additionally, the six asphalt binders were sourced from a single supplier, and the aggregate properties (angularity, texture) were not systematically varied. The simplification of fine aggregates smaller than 2.36 mm as spherical mastic particles may not fully capture their contribution to inter-particle friction and mixing resistance at the micro-scale. The current model does not explicitly simulate polymer chain formation or molecular-level morphological changes of SBS modifiers; instead, these effects are implicitly incorporated through calibrated contact parameters. Future research should address these limitations by (i) integrating temperature-dependent viscoelastic contact models (e.g., the Burgers model) into the DEM framework to explicitly represent binder rheology; (ii) conducting independent validation of the calibrated DEM model against additional binder-gradation combinations; (iii) expanding the material matrix to include a wider range of binder sources and aggregate types; (iv) investigating the influence of fine aggregate morphology on mixing resistance through higher-resolution particle representation; (v) extending this methodology to warm-mix asphalt (WMA) mixtures, where chemical or organic additives are used to reduce mixing temperatures; and (vi) incorporating imaging-based techniques, such as X-ray computed tomography, to validate the DEM-simulated aggregate displacement and contact force distributions.

## 5. Conclusions

This study investigated the macroscopic and mesoscopic mixing workability (MW) of polymer-modified asphalt mixtures (PMAMs) through an integrated experimental and three-dimensional discrete element modeling (PFC3D) approach. The primary contribution of this work is the establishment of a quantitative micro–macro linkage framework that connects mesoscopic DEM contact mechanics to macroscopic torque-based workability evaluation, thereby addressing a critical gap where previous workability studies have relied on phenomenological descriptions without mesoscopic mechanistic underpinning. Based on the results obtained, the following conclusions are drawn:

(1) Temperature exerts the dominant influence on the mixing workability of asphalt mixtures, followed by gradation and asphalt binder type. The sensitivity analysis reveals that torque values increase by up to 115% when the temperature decreases from 165 °C to 90 °C, highlighting the critical importance of strict temperature control during construction.
(2) Polymer-modified asphalt mixtures (PMAMs) exhibit significantly poorer mixing workability than base asphalt mixtures (BAMs) at equivalent temperatures, attributable to the higher binder viscosity and enhanced viscous resistance between aggregates. The torque values of PMAMs are 18–22% higher than those of BAMs under comparable conditions.
(3) Mixtures with larger nominal maximum aggregate size (NMAS) and open gradation exhibit higher torque values (worse MW), primarily due to increased friction resistance and interlock forces among coarse aggregates. Conversely, gap-graded mixtures (SMA-13) show relatively better workability owing to reduced paddle-mixture contact area.
(4) PFC3D simulations reveal that the density of contact force chains and tensile stress increase notably with decreasing temperature, providing mesoscopic evidence for the temperature-dependent deterioration of mixing workability observed experimentally.
(5) Gradation appears to primarily provide a load-transfer pathway without significantly altering the fundamental contact force characteristics (tension vs. compression ratio). However, mixtures with open and gap gradations exhibit larger aggregate displacements during mixing, suggesting better aggregate mobility despite higher torque values.

The primary limitation of this study is that the PFC3D model employs a simplified parallel bond contact model with temperature-dependent parameters calibrated against macroscopic torque data, rather than explicitly incorporating binder rheology as a function of temperature. Future research should (i) integrate temperature-dependent viscoelastic contact models (e.g., the Burgers model) into the DEM framework; (ii) validate the simulation results against field-scale mixing data; and (iii) extend the methodology to warm-mix and recycled asphalt mixtures to assess broader applicability.

## Figures and Tables

**Table 1:** Technical parameters of the digital display torquemeter.

Parameters	Value	Parameters	Value
Type	TWS-50	Square Tenon	3/8”DR
Test Range (N·m)	10 to 60	Accuracy (%)	±0.35
Temperature Range (°C)	−10 to 60	Length (mm)	350

**Table 2:** Fitting values of PFC parameters of SBSM asphalt mixture.

Parameters	*pb_k_n_* (N/m)	*pb_k_s_* (N/m)	*k_n_* (N/m)	*k_s_* (N/m)	*pb_coh* (N)	*pb_ten* (N)	*emod*
90 °C	2.63 × 10^8^	2.63 × 10^8^	1.87 × 10^6^	1.87 × 10^6^	3.01 × 10^4^	3.01 × 10^4^	1.91 × 10^5^
105 °C	2.37 × 10^8^	2.37 × 10^8^	1.75 × 10^6^	1.75 × 10^6^	2.16 × 10^4^	2.16 × 10^4^	1.61 × 10^5^
135 °C	2.03 × 10^8^	2.03 × 10^8^	1.44 × 10^6^	1.44 × 10^6^	1.51 × 10^4^	1.51 × 10^4^	1.35 × 10^5^
165 °C	1.56 × 10^8^	1.56 × 10^8^	1.07 × 10^6^	1.07 × 10^6^	1.03 × 10^4^	1.03 × 10^4^	1.08× 10^5^

**Table 3:** The viscosity of asphalts under the Brookfield viscometer (Pa·s).

Temperature (°C)	AH-70	AH-50	SBSM	SEM	SEPM	HVM
90	9.29	13.11	41.57	34.4	73.42	81.83
105	3.65	4.59	14.23	11.0	27.3	37.1
120	1.40	1.94	5.79	5.07	8.62	11.9
135	0.77	0.96	2.40	2.00	3.90	4.08
150	0.31	0.53	1.01	0.98	1.76	1.93
165	0.15	0.3	0.7	0.55	0.87	1.01

Note: AH-70, AH-50, SBSM, SEM, SEPM, and HVM are abbreviations for 70# penetration-grade base asphalt, 50# penetration-grade base asphalt, SBS-modified asphalt, SEBS-modified asphalt, SEBS- and PPA-compound-modified asphalts, and high-viscosity-modified asphalt, respectively.

**Table 4:** Gradation of asphalt mixture.

**Gradation**	**Passing Mass Percentage (%) of a Certain Sieve Size (mm)**												
	31.5	26.5	19	16	13.2	9.5	4.75	2.36	1.18	0.6	0.3	0.15	0.075
ATB-25	100	95.5	70.9	59.9	53	44.8	30.7	22.4	17.5	12.2	8.5	5.8	4.9
AC-20	-	100	95	85	71	61	41	30	22.5	16	11	8.5	5
AC-13	-	-	-	100	95	76.5	53	37	26.5	19	13.5	10	6
OGFC-13	-	-	-	100	95	70	21	16	12	9.5	7.5	5.5	4
SMA-13	-	-	-	100	95	62.5	27	20.5	19	16	13	12	10

Note: ATB, AC, OGFC, and SMA represent open-graded asphalt stabilized macadam, dense-graded asphalt concrete, open-graded friction course asphalt mixture, and stone mastic asphalt, respectively. The number following each abbreviation indicates the nominal maximum aggregate size.

**Table 5:** Sensitivity analysis results.

Factors	Basic	Comparison	Difference (N·m)	Percentage (%)	Sensitivity Grade
Asphalt	AH-70	SEM	1.24	+7.20	Low
		SBSM	1.94	+11.27	Low
		SEPM	3.16	+18.35	Medium
		HVM	3.85	+22.36	Medium
Temperature	165 °C	170 °C	−0.85	−4.94	Low
		150 °C	3.26	+18.93	Medium
		135 °C	5.76	+33.45	Medium
		90 °C	19.89	+115.51	High
Gradation	AC-13	ATB-25	4.01	+23.29	Medium
		AC-20	3.15	+18.29	Medium

Note: The sensitivity classification is based on the relative percentage change in torque with respect to the reference condition (AC-13 with AH-70 at 165 °C), not on an assumption of physical equivalence between the factor ranges. The percentages represent the proportional torque change caused by varying each factor from its reference level to the comparison level. The critical thresholds of 15% and 50% were selected to provide practical guidance for construction quality control: a 15% change in torque corresponds to a noticeable but manageable workability difference, while a 50% change represents a substantial operational challenge. The purpose of this analysis is to prioritize quality control measures at construction sites, not to compare the absolute magnitudes of physically different variables.

## Data Availability

The original contributions presented in this study are included in the article. Further inquiries can be directed to the corresponding authors.
